# Pain Expressions in Dementia: Validity of Observers’ Pain Judgments as a Function of Angle of Observation

**DOI:** 10.1007/s10919-019-00303-4

**Published:** 2019-03-21

**Authors:** M. Erin Browne, Thomas Hadjistavropoulos, Kenneth Prkachin, Ahmed Ashraf, Babak Taati

**Affiliations:** 1 Department of Psychology and Centre on Aging and Health, University of Regina, Regina, SK Canada; 2 Department of Psychology, University of Northern British Columbia, Prince George, BC Canada; 3 Toronto Rehabilitation Institute, University of Toronto, Toronto, ON Canada; 4 Institute of Biomaterials and Biomedical Engineering and Department of Computer Science, University of Toronto, Toronto, ON Canada

**Keywords:** Nonverbal expression, PACSLAC-II, Behavioral assessment, Older adults, Elderly

## Abstract

Facial expressions of pain are important in assessing individuals with dementia and severe communicative limitations. Though frontal views of the face are assumed to allow for the most valid and reliable observational assessments, the impact of viewing angle is unknown. We video-recorded older adults with and without dementia using cameras capturing different observational angles (e.g., front vs. profile view) both during a physiotherapy examination designed to identify painful areas and during a baseline period. Facial responses were coded using the fine-grained Facial Action Coding System, as well as a systematic clinical observation method. Coding was conducted separately for panoramic (incorporating left, right, and front views), and a profile view of the face. Untrained observers also judged the videos in a laboratory setting. Trained coder reliability was satisfactory for both the profile and panoramic view. Untrained observer judgments from a profile view were substantially more accurate compared to the front view and accounted for more variance in differentiating non-painful from painful situations. The findings add specificity to the communications models of pain (clarifying factors influencing observers’ ability to decode pain messages). Perhaps more importantly, the findings have implications for the development of computer vision algorithms and vision technologies designed to monitor and interpret facial expressions in a pain context. That is, the performance of such automated systems is heavily influenced by how reliably these human annotations could be provided and, hence, evaluation of human observers’ reliability, from multiple angles of observation, has implications for machine learning development efforts.

## Introduction

Nonverbal expressions of pain (e.g., in response to acute and phasic pain exacerbations due to movement) are important in all forms of pain assessment because, compared to self-report, they are less likely to be influenced by situational demand characteristics and cognitive executive mediation (e.g., Craig et al. 2001; Hadjistavropoulos et al. 2011). The evaluation of such expressions is vital in situations where the patient has limited ability to communicate verbally due to severe cognitive impairment related to dementia (e.g., Hadjistavropoulos et al. 2014; Herr et al. 2011). As a result, a variety of methods have been validated with respect to their ability to detect the intensity of experienced pain in dementia (Aubin et al. 2007; Herr 2011; Husebo et al. 2010; Kunz et al. 2007; Lints-Martindale et al. 2007, 2012).

It is widely assumed that judgments of others’ facial expressions of pain are optimized when the observations are based on full, dynamic views of the face. These views typically include at least the front view of the face, and sometimes also profile views as they occur during in vivo observation. To the best of our knowledge, however, there has never been a study that has confirmed this assumption. This question is of theoretical importance because it addresses a factor that could impact pain communication and influence an observer’s ability to decode pain expressions (see Hadjistavropoulos et al. 2011). Moreover, in a clinical context, staff members often observe patients from profile views (e.g., in cases where patients are lying in a hospital bed or sitting on a chair in a common area) but the impact of different angles of observation on pain assessment is not known.

The nature of decoding pain expressions from different angles of observation is also important from the perspective of computerized vision system development. Computer vision systems are being developed to monitor pain behaviors in older adults with dementia (Ashraf et al. 2009, 2016). Efforts include adaptation of these systems so that they can identify pain behaviors as older adults go about their regular routines. From a practical standpoint, such systems would need to recognize pain expressions from multiple angles of observation including profile views. Algorithms currently used for pain expression recognition rely on information from human observers/coders. Testing the extent to which human coders and observers can accurately decode pain expressions from a profile view will help to determine the feasibility of profile-view pain expression computer algorithms. We note that machine learning models can sometimes reach super-human performance, even when they are trained on human-annotated data (Bartlett et al. 2014; e.g., by learning to ignore erroneous annotations that do not fit a general pattern). Nevertheless, the performance of such automated systems is heavily influenced by how reliably these human annotations could be provided and, hence, evaluation of human observers’ reliability, from multiple angles of observation, has implications for machine learning development efforts. Within the context of a broader investigation, we video recorded patients during a discomforting physiotherapy examination (Hadjistavropoulos et al. 2018). Recordings were made simultaneously from different angles of observation (i.e., front view, with the camera overhead as patients were lying on their back, as well as profile views). Our goal was to examine the relative validity and reliability of observations based on a panoramic view of the face (including profile views and front views) to observations made based on profile view alone.

This investigation was completed in two parts. In “Study 1”, we investigated interrater reliability between trained coders using two established methods for behavioral pain coding during panoramic view coding and during profile coding: (a) a modified version of the Facial Action Coding System (FACS; Ekman and Friesen 1978); and (b) the Pain Assessment Checklist for Seniors with Limited Ability to Communicate-II (PASCSLAC-II; Chan et al. 2014). We also examined the ability of trained coders to discriminate between painful versus non-painful situations based on the full versus profile view of the face using a FACS-based approach and the PACSLAC-II. In “Study 2”, we investigated untrained observers’ ability to differentiate baseline video segments from discomforting examination segments as well as observer judgmental accuracy (i.e., the correspondence of observers’ pain intensity judgements with the validated and standardized FACS-based system and the PACSLAC-II) for pain intensity using the panoramic versus the profile view of the face. Through lens model analysis, we also examined the cues that trained coders and untrained observers utilized in making their pain intensity judgments.

## Study 1

### Method

#### Participants

As part of a larger investigation (see Hadjistavropoulos et al. 2018) we recruited 102 adults older than 65 years of age (*M* = 78.84, *SD* = 8.51). Forty-eight (13 male) of our participants were recruited from local long-term care facilities after identification by facility care staff as having dementia and severe limitations in verbal communicative ability. Fifty-two participants (19 male) living independently in the community were recruited through a physiotherapy clinic where they were being treated. Potential participants were excluded if they had known acute pain problems such as fractures.

For long-term care participants, proxy consent was obtained by family members or legal guardians (i.e., the same individuals who would normally provide consent for medical procedures on behalf of residents with dementia). Those providing proxy consent were given an information package as well as the opportunity to ask questions about the study and its voluntary nature. Patient assent was sought in all cases, and no patient took part if he or she gave any verbal and behavioral indications of unwillingness to take part in the task. As a token of appreciation for their participation, $20 was set aside for each resident to purchase items that, according to caregivers or legal guardians, the patient would enjoy (e.g., flowers, music CDs, photo frames). The final long-term care sample had a Cognitive Performance Scale (CPS) Score (Morris et al. 1994) of 3.74 (*SD* = 1.72) which placed them in the moderate to severe range of dementia. These scores were obtained from the patients’ charts and were never older than 3 months. Potential community participants were excluded if they were not independent (i.e., if they could not attend appointments unaccompanied). Eligible community participants were made aware of the study by clinic staff and, if interested, given the information package and an opportunity to ask questions about the study. They were offered $20 for their participation.

#### Measures

##### Facial Action Coding System

The Facial Action Coding System (FACS) is an objective measure of facial activity, which allows for coding of 44 discrete movements (and combinations thereof) of underlying facial musculature in terms of frequency (present or not present) and intensity (0–5; Ekman and Friesen 1978). These movements, referred to as action units (AUs; e.g., cheek raising, lip raising, brow furrowing), are evaluated by trained coders, and certain combinations of such movements have been found to correspond to prototypical facial expressions of emotion (e.g., sadness, disgust, surprise; Ekman and Friesen 1978) as well as pain (Prkachin and Solomon 2008). The FACS has been shown to be highly reliable and has been comprehensively validated in investigations of nonverbal pain behavior in a variety of populations including older adults with dementia (Craig et al. 2001; Hadjistavropoulos et al. 2014; Lints-Martindale et al. 2007). Certain AUs have been reliably associated with facial expressions during painful experiences, including brow lowering (AU4), cheek raising (AU6), lid tightening (AU7), nose wrinkling (AU9), upper lip raising, (AU10), and closing of the eyes (AU43) (Prkachin 1992; Prkachin & Solomon 2008). Other facial actions, in particular nasolabial furrow deepening (AU 11), lip corner pull (AU 12,) lip stretch (AU 20), varying degrees of mouth/jaw opening (AUs 25, 26, 27), and blinking (AU 45) have occasionally been found to be associated with pain in previous research. Some of these associations have not been reported frequently (e.g., AU 11; AU 20), possibly due to the rarity with which they occur. Others (e.g., AUs 12, 25, 26, 27) also occur less reliably in association with pain and, when they do, appear to be part of a different reaction (Kunz et al. 2013; Prkachin and Solomon 2008). Similarly, when AU 45 occurs in association with pain, it appears to relate to the properties of the pain modality that give rise to startle (Craig and Patrick 1985; Prkachin 1992). In this study, we restricted attention to the actions most frequently and reliably associated with pain in existing research.

##### Pain Assessment Checklist for Seniors with Limited Ability to Communicate-II

The Pain Assessment Checklist for Seniors with Limited Ability to Communicate-II (PACSLAC-II; Chan et al. 2014) is an easy to use and validated observational assessment tool developed for use by health professionals in clinical settings (Chan et al. 2014; Hadjistavropoulos et al. 2018). The PACSLAC-II consists of a checklist of 31 pain behaviors (i.e., verbalizations, body movements, facial expressions, changes in activity, interpersonal changes, mental status changes) corresponding to recommended domains of nonverbal pain assessment set out by the American Geriatrics Society (AGS) Panel on Persistent Pain in Older Persons (2002). The PACSLAC-II items are classified under specific subscales: verbalizations and vocalizations, facial expressions, body movements, changes in interpersonal interactions, changes in activity patterns or routines, and mental status changes. Each pain behavior is coded as present or not present (0 or 1) in a given interaction, resulting in a maximum nonverbal pain behavior score of 31 on the PACSLAC-II. The PACSLAC-II has been found to be valid, reliable, and discriminates between painful and non-painful situations (Chan et al. 2014; Ammaturo et al. 2017). For the purposes of “Study 1”, the full PACSLAC-II was used by trained coders to evaluate videos of older adults during baseline and painful situations. In “Study 2”, videos were edited to only include the face of the older adult (therefore removing postural and body movement information), and audio was removed. Thus, only the Facial Expressions subscale of the PACSLAC-II was appropriate for use as a point of comparison to observer judgments in “Study 2”; analyses reflect this. The PACSLAC-II Facial Expressions subscale includes 11 items, coded as present or not present (0 or 1): grimacing, tighter face, pain expression, increased eye movement, wincing, opening mouth, creasing forehead, lowered eyebrows or frowning, raised cheeks, narrowing of the eyes or squinting, wrinkled nose and raised upper lip, and eyes closing. Thus, the maximum PACSLAC-II Facial Expressions nonverbal pain behavior score is 11.

#### Procedure

Video data were collected in long-term care facilities and in an outpatient physiotherapy clinic. Participants were video recorded lying on a bed during a 5-min still baseline period, and while they engaged in a movement-based physiotherapy examination designed to identify painful areas. The examination has been described by Husebo and colleagues and used in previous investigations of movement exacerbated pain (Husebo et al. 2007, 2008, 2010). Specifically, following the baseline period, each participant remained lying down on a bed or examination table. A physiotherapist then guided the patient to move one limb at a time, as follows: close each hand, raise each arm, bend each knee toward the chest, extend each foot, and sit up on the bed or examination table. The movement phase took approximately 5 min. All community participants were capable of self-reporting pain using a 0–10 numeric rating scale (baseline mean pain rating = 1.83, *SD* = 2.25; pain examination mean rating = 5.45, *SD* = 2.61). Participants’ faces were filmed from the front and profile views simultaneously during the baseline and movement phases. We note that there was a brief moment during the protocol that participants facial expression could not be captured by the camera (as they were getting up to sit on the side of the bed or examination table).

Following collection of video data, coding was completed for the entirety of each participant’s baseline and pain videos with a panoramic view using a previously validated FACS-based approach for pain evaluation (Gagnon et al. 2017; Gallant and Hadjistavropoulos 2017; Prkachin and Solomon 2008) as well as the PASCLAC-II. The modified FACS coding approach, originally described by Prkachin and Solomon (2008), involved four categories of nonverbal pain-related facial actions: (1) brow lowering (i.e., AU4); (2) orbit tightening (i.e., AU6 and AU7); (3) levator tightening (i.e., AU9 and AU10); and (4) closing of the eye (i.e., AU43). The index combines information from the six actions that have been reliably associated with pain across modalities and populations studied in previous research (Kunz et al. 2018). For each video frame, a maximum intensity score was assigned for brow lowering, orbit tightening, and levator tightening using a 0 (i.e., *no facial action*) to 5 (i.e., *maximum facial action*) scale. Closing of the eyes was scored as either 0 (*not present*) or 1 (*present*). The maximum intensity score for each category was summed to create a nonverbal pain expression score (i.e., ranging from 0 to 16) for each video frame.

A different trained coder then completed the same set of coding for each participant, using only a profile view of the face (left side). Trained coders completed frame-by-frame coding, using Noldus ObserverXT software (Noldus Information Technology 2015); this approach results in approximately 5000 frames per baseline and movement segment, and is far more rigorous than what is typically conducted in facial expression research. This frame-by-frame coding approach was necessary to facilitate the creation of machine learning algorithms, for which the video dataset will also be used.

Reliability was assessed using the Noldus ObserverXT analysis module. Reliability of panoramic-view FACS-based and PACSLAC-II coding was assessed systematically over the course of data collection by random selection of recordings of baseline and physiotherapy examination sessions from 21 participants, comprising 17 baseline and 21 test sessions. For each selected recording, two proficient coders independently coded the same video. For profile coding reliability, two proficient coders independently coded 15 randomly selected profile-view videos.

#### Analysis

Reliability analyses were conducted to determine trained coder agreement using FACS-based (Pearson’s rho coefficient, which allows for examination of modifiers such as 0–5 intensity coding) and PACSLAC-II (kappa coefficient, which allows for examination of dichotomous items) coding, from each viewing angle and during each procedure segment (baseline vs. examination). To investigate differences in ability of trained coders to discriminate the pain-related movements from the baseline using pain behaviors among we conducted two 2 (baseline vs. physiotherapy examination) × 2 (panoramic vs. profile view) analyses of variance (ANOVAs) using FACS-based and PACSLAC-II scores as dependent measures. Any significant interactions were followed up using pairwise comparisons.

### Results

**Fig. 1:**
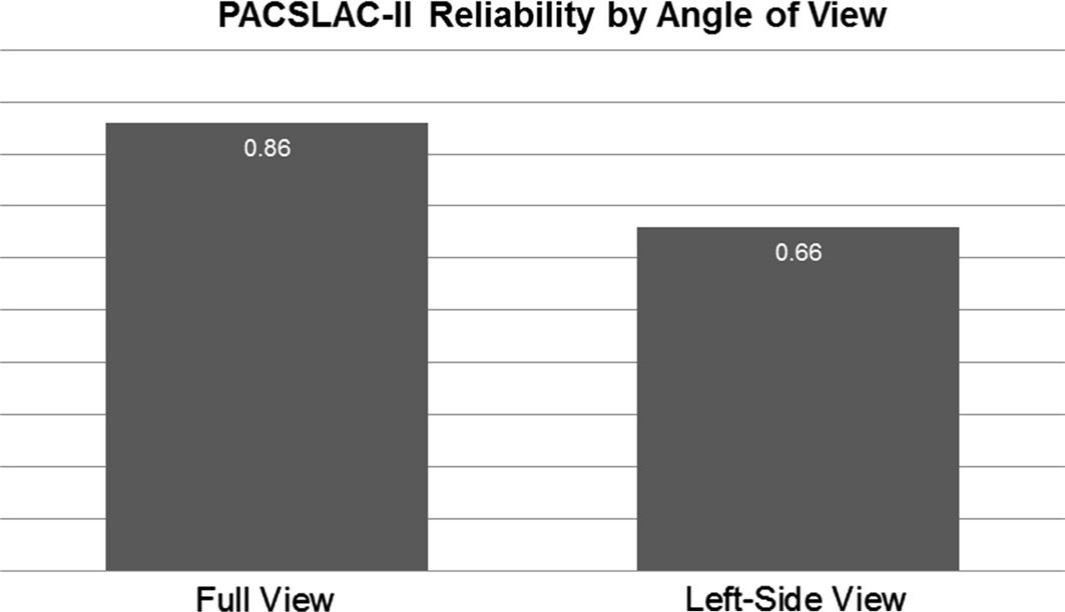
PACSLAC-II reliability by angle of view. Reliability in this graph refers to kappa values. *PACSLAC-II* pain assessment checklist for seniors with limited ability to communicate-II

**Fig. 2:**
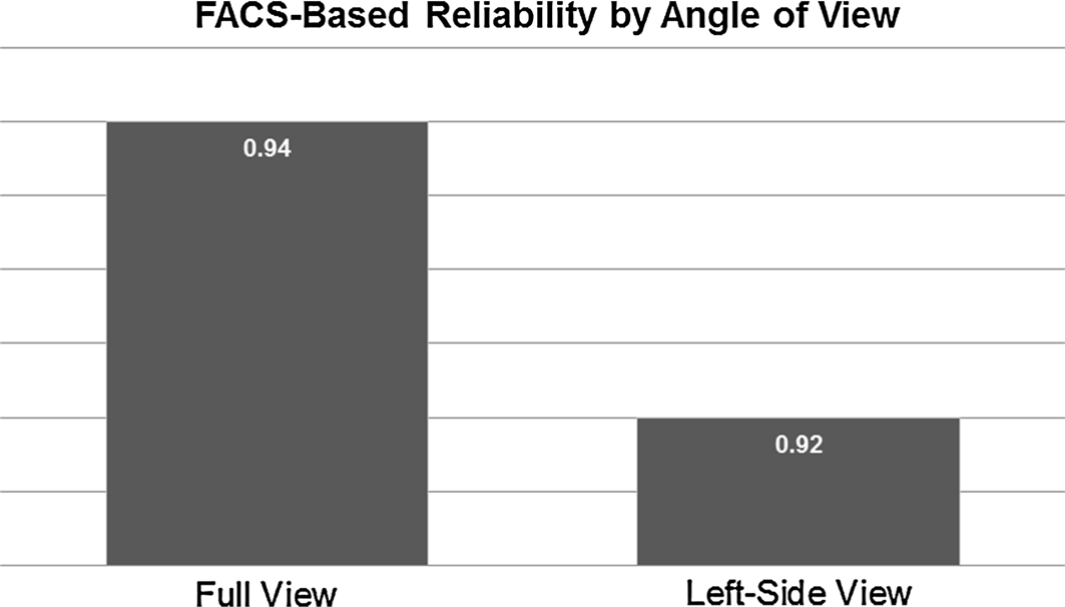
FACS-based reliability by angle of view. Reliability in this graph refers to rho values. FACS-based = modified coding for pain based on the Facial Action Coding System

Reliability was calculated for each coding system during baseline and physiotherapy examination segments, from each angle of observation, and with each pain rating system. Pearson’s rho for FACS-based coding and kappa for PACSLAC-II coding are presented in Table 1. Reliability of PACSLAC-II coding was somewhat higher using the panoramic rather than the profile view (Fig. 1); FACS-based reliability was very similar using the full and profile views (Fig. 2).

**Table 1:** Interrater reliability across coding systems and viewing angles

	FACS-based (ρ)		PACSLAC-II (κ)	
	Baseline	Exam	Baseline	Exam
Full view	.99	.94	.92	.86
Profile view	.96	.92	.81	.66

Table 2 shows the means and standard deviations of trained observers on FACS-based and PACSLAC-II coding. Two 2 (baseline vs. physiotherapy examination) x 2 (panoramic vs. profile view) ANOVAs were conducted using FACS-based and PACSLAC-II scores as dependent measures. As anticipated, a significant main effect confirmed that trained coders identified more pain behavior during the physiotherapy than during the baseline using the FACS-based score, *F*(1100) = 38.368, *p* < .001, partial *η*^2^ = .277. There was no significant within-subjects effect of angle of observation on FACS-based scores and the interaction effect was also not significant. The second ANOVA involved trained coder PACSLAC-II scores as dependent measure, and revealed a significant main effect for video segment (i.e., baseline vs. examination), *F*(1100) = 120.437, *p* < .001, partial *η*^2^ = .546, indicating that trained observers judged individuals as being in more pain during the physiotherapy examination compared to the during baseline. There were no other significant effects.

**Table 2:** Descriptive statistics for trained observer ratings

	FACS-based				PACSLAC-II			
	Baseline		Examination		Baseline		Examination	
	*M*	*SD*	*M*	*SD*	*M*	*SD*	*M*	*SD*
Full view	^a^2.55	1.92	^a^4.32	2.29	^a^3.12	1.85	^a^7.16	3.37
Profile view	^a^2.55	1.92	^a^4.24	2.32	^a^3.15	1.83	^a^7.11	3.44

^a^Denotes significant difference in ratings by segment (baseline vs. examination)

## Study 2

### Method

#### Procedure

Using trained coder judgments as the criterion (modified FACS and PACSLAC-II, described in the “Study 1” section), we examined the accuracy of untrained observers’ pain intensity judgments based on the profile view of the face versus the panoramic view, during baseline and physiotherapy examination.

Sixty-one undergraduate students (mean age = 22.72, *SD* = 4.45; 41 females) viewed 80 randomized-order 10-s segments of older adults (65 +) with and without dementia undergoing a physiotherapy examination. All stimulus video segments were taken from recordings made in “Study 1”. Stimulus video segments were chosen based on the following criteria, in order of consideration: optimal video quality (i.e., in focus, well-lit, appropriately-angled footage); ensuring a wide range of pain expressions (i.e., 0–16 pain intensity as per FACS-based coding); sex of older adult (i.e., 20 males and 20 females); cognitive status (i.e., 20 participants with severe dementia, and 20 without); age of older adult (i.e., a range representative of the dataset); and length of entire judgment task (i.e., under 60 min to avoid observer fatigue). Accommodation of all parameters resulted in a total of 40 older adults appearing in the video segments. Each observer saw each target individual’s face twice: once in a video segment showing a profile view, and once in a video segment showing a frontal view (each view was of the same 10 s of expressed behavior) and the order of all segments was fully randomized. Each segment appeared on the computer screen for its duration, followed by a prompt to make a rating, and a 15-s blank screen. Using a pencil and paper numeric rating scale (0–10), participants made judgments of how much pain each older adult was feeling in each segment.

#### Analysis

To determine whether untrained observers’ pain intensity judgments would be highest during a physiotherapy protocol designed to identify painful areas (as compared to a baseline condition) and to identify if there were any differences in scores based on view, we conducted a within-subjects (Panoramic vs. Profile x Baseline vs. Physiotherapy Examination) analysis of variance (ANOVA) using observer pain intensity judgment scores as dependent measure. Any significant interaction effects were followed up using pairwise comparisons. Possible gender differences in the expression and judgment of pain intensity were explored using one-way ANOVAs.

Untrained observer judgmental accuracy was defined as correspondence between untrained coder pain intensity judgments and two criterion indices: trained coder FACS-based coding and trained coder PACSLAC-II Facial Expression subscale coding. We calculated difference scores based on standardized observer pain ratings and trained coder FACS-based and PACSLAC-II scores. In addition, two correlation coefficients were calculated for each untrained observer to assess accuracy against a criterion (i.e., the degree of correspondence between untrained observer judgments and FACS-based coding, and the degree of correspondence between untrained observer judgments and PACSLAC-II coding). All correlation coefficients were transformed using Fisher’s *r* to *z* transformation before they were used in subsequent analyses. One sample *t*-tests determined if accuracy differed from zero. Paired-samples *t*-tests were used to identify differences in untrained observer coding accuracy (as defined by correspondence with each coding system) from a panoramic versus profile view.

To examine possible differences in cues attended to by trained and untrained observers from each view, lens model analyses (Brunswik 1952) were performed for each coding system. The lens model analysis allows for direct investigation of the utilization of each behavioral cue (e.g., mouth open, frown) by trained and untrained observers in making overall judgments of pain intensity. To do so, the association (measured with Pearson’s *r*) between each cue (e.g., frown or brow lowering) and overall pain intensity judgment is calculated for each group of judges. Thus, it is possible to determine the relative weight that trained versus untrained observers place on each behavioral cue when making a pain judgment by comparing the correlation coefficient between each cue and overall pain intensity of the trained coders versus untrained judges. Similar comparisons were made regarding cue utilization within the same group of judges from each view (e.g., to determine whether trained judges rely on the same cues from the panoramic vs. profile view). To evaluate differences between trained and untrained observer cue utilization, Steiger’s *Z* statistic for overlapping dependent correlations was utilized (Steiger 1980). To evaluate differences in cue utilization within judge groups from the panoramic versus profile views, the Pearson–Filon *Z* test for non-overlapping dependent correlations was used (Pearson and Filon 1898).

For PACSLAC-II coding lens model analysis, both panoramic and side view models included the following cues: P1 (grimacing), P2 (tighter face), P3 (pain expression), P5 (wincing), P6 (opening mouth), P7 (creasing forehead), P8 (brow lowering or frowning), P9 (raising cheeks, narrowing eyes or squinting), P10 (wrinkled nose), and P11 (eyes closing). P4 (increased eye movement) was not observed in “Study 1”, and so was not considered in the lens model. For FACS-based coding lens model analysis, the following cues were included: brow lowering, orbit tightening, levator activation, and eyes closing.

### Results

#### Observers’ Accuracy and Ability to Differentiate Baseline from Pain Expressions

**Fig. 3:**
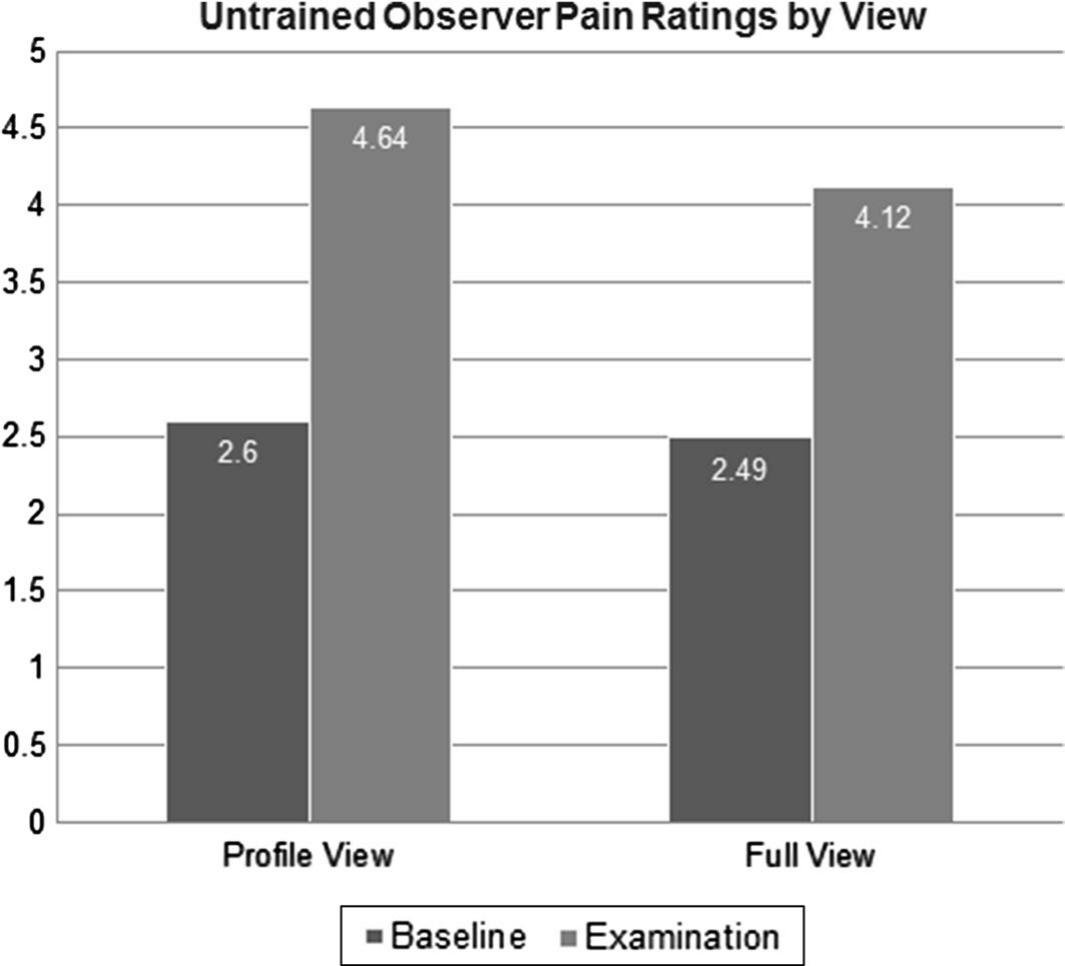
Untrained observers’ differentiation of pain (examination) from no-pain (baseline) segments

**Fig. 4:**
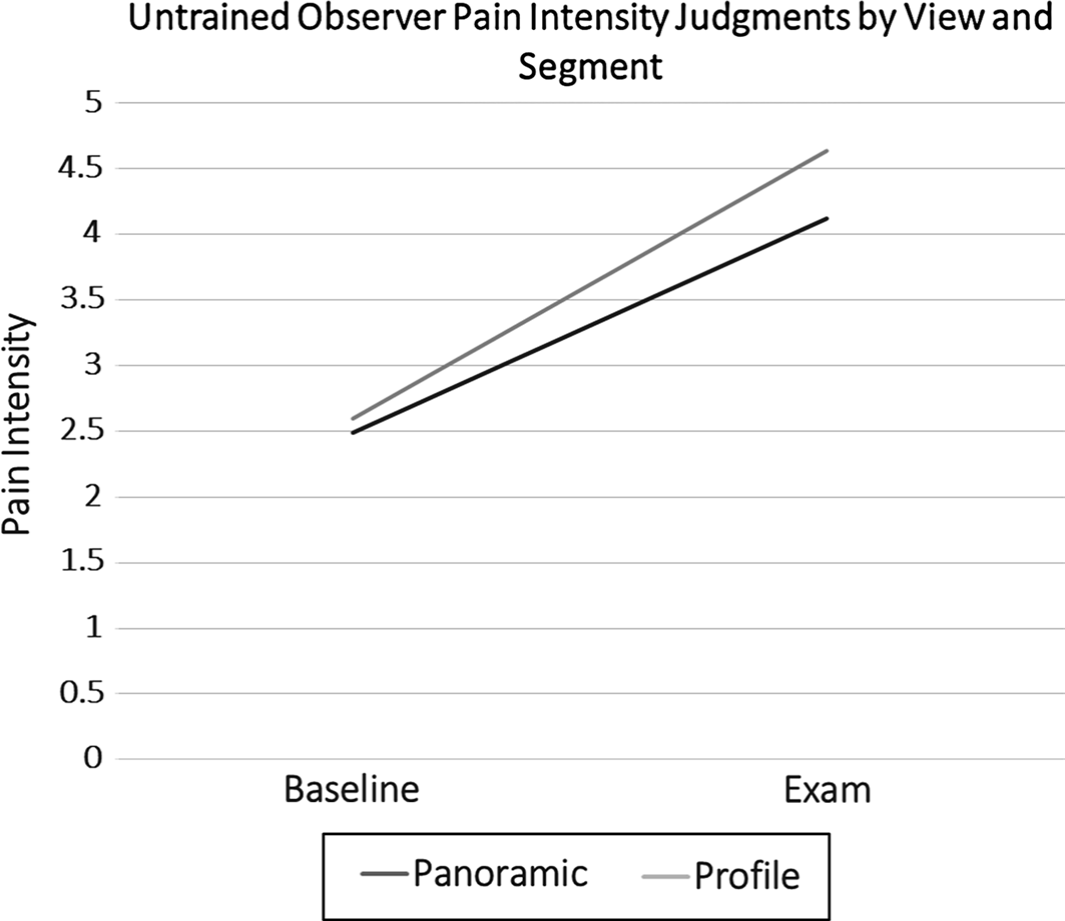
Interaction effect of segment and view (untrained observer pain intensity ratings)

Table 3 shows the means and standard deviations of untrained observers’ pain intensity judgments. As anticipated, the within subjects ANOVA (Panoramic vs. Profile × Baseline vs. Physiotherapy Examination) resulted in a significant main effect, which confirmed that untrained coders identified more pain behavior during the physiotherapy examination than the baseline, *F*(1100) = 289.455, *p* < .001, partial *η*^2^= .828 (Fig. 3). There was a significant within-subjects effect of angle of observation on observer pain intensity judgement scores, *F*(1100) = 27.433, *p* < .001, partial *η*^2^= .314. The interaction effect was also significant, *F*(1100) = 29.819, *p* < .001, partial *η*^2^= .332. Follow-up analysis of this interaction indicated that there was a significant difference in observer pain intensity judgment scores between panoramic and profile view during the examination (Mean Diff = .522, *SE *= .069. *p *< .001), but not during baseline (Fig. 4). This indicated that untrained observers judged pain intensity as being higher from the profile view (*M *= 1.80, *SD* = 1.38) than from the panoramic view (*M* = 1.60, *SD* = 1.30) during the examination. Variances were not significantly different between views during baseline (panoramic *SE* = .187; profile *SE* = .186) and exam (panoramic *SE* = .167, profile *SE* = .176).

**Table 3:** Descriptive statistics for untrained observer ratings

	Pain ratings			
	Baseline		Examination	
	*M*	*SD*	*M*	*SD*
Full view	2.49^a^	1.46	4.12^ab^	1.30
Profile view	2.60^a^	1.46	4.64^ab^	1.38

^a^Denotes significant difference in ratings by segment (baseline vs. examination)

^b^Denotes significant difference in ratings by view (full vs. profile)

We conducted one-way analyses of variance with observer gender as factor, investigating differences between mean pain ratings from the profile and panoramic views, during baseline and exam segments. We also conducted a parallel analysis of variance using stimulus patient gender as factor and overall expressiveness as dependent. There were no significant gender differences in judgments or expression.

Table 4 shows difference and accuracy scores based on standardized observer pain ratings and trained coder FACS-based and PACSLAC-II scores. These scores suggest that trained observers tended to rate pain lower than untrained observers. A more precise measure of untrained observers’ accuracy was developed by directly examining the relationship between their pain intensity ratings and the FACS-based and PACSLAC measurements. Pearson correlation coefficients were calculated between observers’ pain intensity ratings and the trained coder FACS-based and PACSLAC-II measurements at the level of the individual observer. This generated two accuracy indices per observer (one corresponding to FACS-based pain ratings, and one corresponding to PACSLAC-II Facial Expression subscale ratings). A series of one-sample *t*-tests was conducted to examine if accuracy was significantly higher than zero. All of the one sample *t*-tests yielded significant results, suggesting that accuracy was higher than zero. These results were as follows: profile view PACSLAC-II facial expression accuracy, *t*(60) = 21.397, Mean Difference (*MD)* = .576, *p* < .001, *d* = 2.74; frontal view PACSLAC-II facial expression accuracy, *t*(60) = 14.4, *MD* = .431, *p* < .001, *d* = 1.84; profile view FACS-based accuracy, *t*(60) = 19.245, *MD* = .647, *p* < .001, *d* = 2.46; and frontal view FACS-based accuracy, *t*(60) = 14.552, *MD* = .400, *p* < .001, *d* = 1.86.

**Table 4:** Difference and accuracy scores between trained versus untrained observers

	*r*	*MDiff*
PACSLAC-II		
Full view	.43	− 2.25
Profile view	.58	− 1.25
FACS-based		
Full view	.40	− 1.30
Profile view	.65	− 2.50

**Fig. 5:**
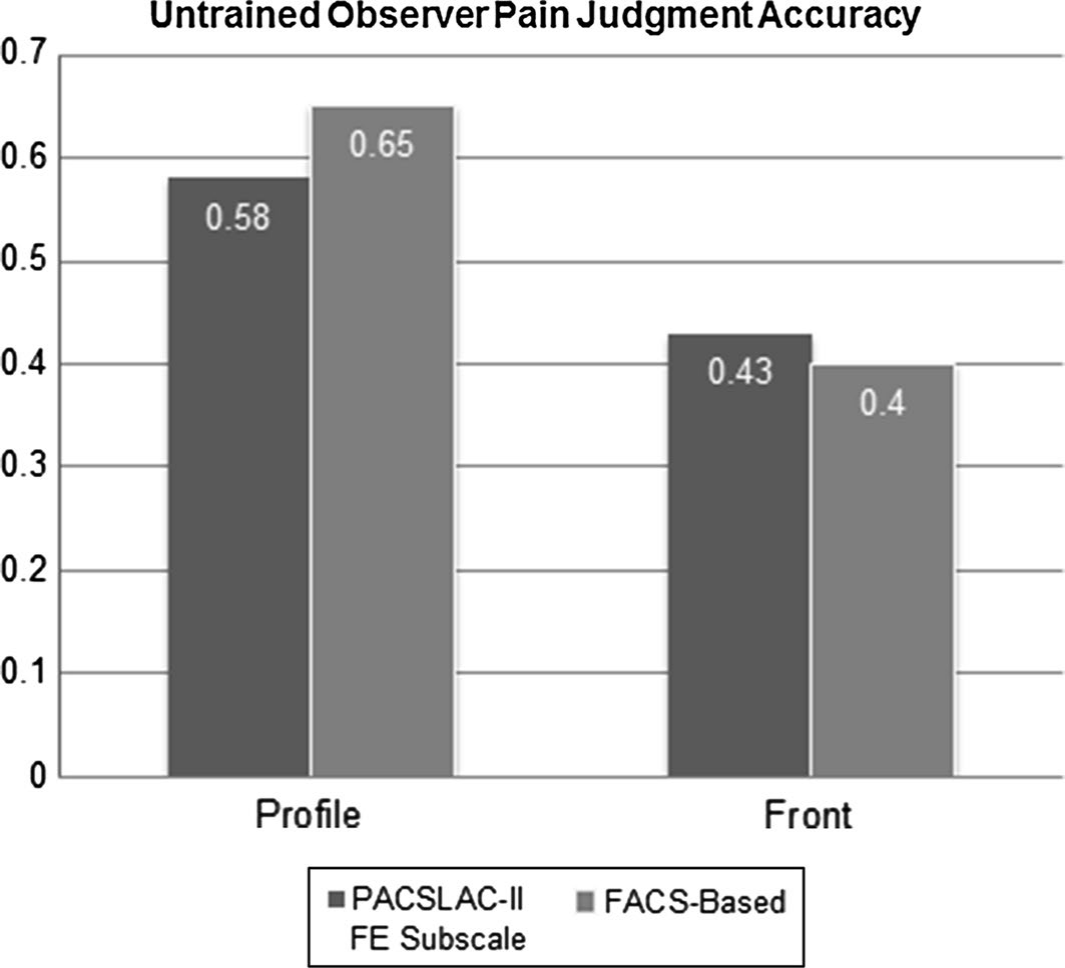
Untrained observers’ pain judgment accuracy. Accuracy in this graph refers to transformed correlation coefficients between observer judgments and expert ratings. *PACSLAC-II* Pain Assessment Checklist for Seniors with Limited Ability to Communicate-II; *FACS-based* modified coding for pain based on the Facial Action Coding System

The accuracy indices were then used as dependent variables in paired samples *t*-tests after Fisher’s *r* to *z* transformation (means are reported as untransformed values below). Paired samples *t*-tests indicated that untrained observers were significantly more accurate in judging pain intensity (i.e., their judgments corresponded to those made by trained pain coders using PACSLAC-II facial expression subscale) from the profile (*M* = .58, *SD* = .21) than from the front view (*M *= .43, *SD* = .23); *t*(60) = 6.559, *p* < .01, *d* = .84. Observers were also significantly more accurate (as compared to FACS-based coding) from the profile (*M* = .65, *SD* = .26) than from the front (*M* = .40, *SD* = .21); *t*(60) = 10.256, *p* < .01, *d* = 1.31. This suggests that when using well-validated systematic observational pain approaches as point of comparison, untrained observers are more accurate when viewing faces from the profile than from the front (FACS-based coding and PACSLAC-II Facial Expression subscale; Fig. 5).

#### Lens model analysis

**Fig. 6:**
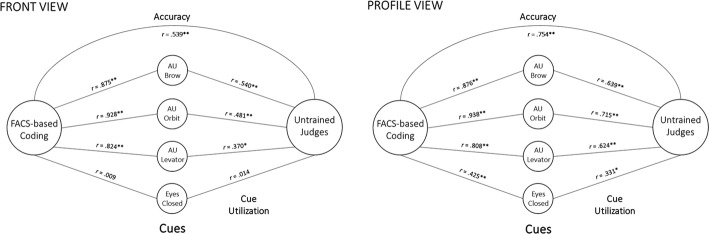
FACS-based lens model analyses

**Fig. 7:**
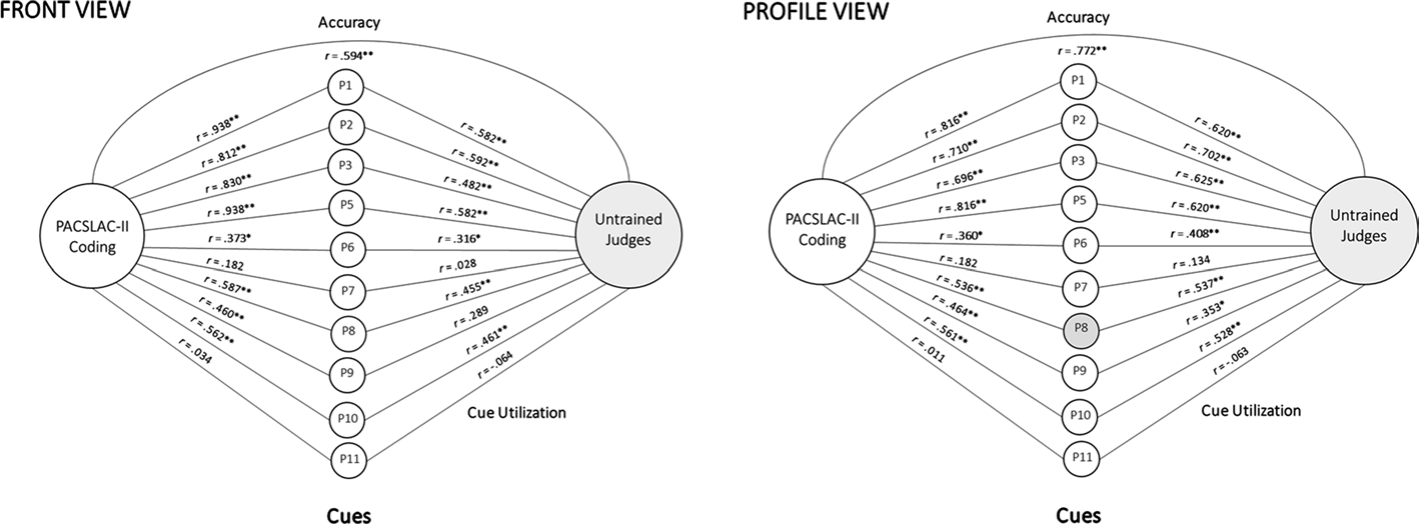
PACSLAC-II lens model analyses

Figure 6 shows the lens model analysis for trained FACS-based coding versus untrained judges’ from a panoramic view as well as from profile view. Figure 7 shows the corresponding analysis for PACSLAC-II coding. The figures illustrate the relative association between each behavioral cue and untrained versus trained pain intensity judgments. To determine whether these associations represent significantly different use of cues in trained versus untrained judges, a series of Steiger’s *Z* tests was performed (Table 5). Considering FACS-based behavioral cues: from both the panoramic and profile views, untrained coders’ judgments were significantly less associated than trained coders’ judgments with brow lowering, orbit tightening, and levator activation (Table 5). Considering PACSLAC-II behavioral cues: from a panoramic view, untrained judgments were significantly less associated than trained coders’ judgments with P1 (grimacing), P2 (tighter face), P3 (pain expression), and P5 (wincing). From the profile view, untrained coders’ judgments were significantly less associated than trained coders’ judgments with P1 (brow lowering) and P5 (wincing).

**Table 5:** Differences between cue utilization in trained and untrained observers

Cue	Panoramic view			Profile view		
	Trained observer *r*	Untrained observer *r*	Steiger’s *Z*	Trained observer r	Untrained observer *r*	Steiger’s *Z*
PACSLAC-II						
P1	**0.938**	**0.582**	− 3.445**	**0.816**	**0.620**	2.807**
P2	**0.812**	**0.592**	2.426*	**0.710**	**0.702**	0.108
P3	**0.830**	**0.482**	3.669**	**0.696**	**0.625**	0.895
P4	**0.938**	**0.582**	5.450**	**0.816**	**0.620**	2.807**
P6	**0.373**	**0.316**	0.416	**0.360**	**0.408**	− 0.473
P7	0.182	0.028	1.049	0.182	0.134	0.439
P8	**0.587**	**0.455**	1.091	**0.536**	**0.537**	− 0.011
P9	**0.460**	0.289	1.272	**0.464**	**0.353**	1.111
P10	**0.562**	**0.461**	0.826	**0.561**	**0.528**	0.363
P11	0.034	− 0.064	0.662	0.011	− 0.063	0.667
FACS-based						
Brow	**0.875**	**0.540**	3.828**	**0.876**	**0.639**	3.73**
Orbit	**0.928**	**0.481**	5.725**	**0.938**	**0.715**	4.691**
Levator	**0.824**	**0.370**	4.248**	**0.808**	**0.624**	2.529*
Eyes closed	0.009	0.014	− 0.032	**0.425**	**0.331**	− 2.100*

Bolded entries are significantly different from zero at the .05 level; ** indicates significance at .01 level; * indicates significance at .05 level

To examine differences in cue utilization within judgment type, Pearson–Filon *Z* tests were performed using associations between behavioral cues from the panoramic versus profile views. Considering FACS-based behavioral cues: eyes closing was significantly associated with both trained and untrained observer judgments from the profile view. This difference in eyes closed cue utilization from panoramic to profile view was significant for trained judges, Pearson–Filon *Z* = − 2.83, *p* = .005, and for untrained judges, Pearson–Filon *Z* = − 1.93, *p* = .05. Untrained judges also relied significantly more on orbit tightening, Pearson–Filon *Z* = − 2.20, *p* = .028, and levator movement, Pearson–Filon *Z* = − 1.95, *p* = .05, when judging from the profile view versus the panoramic view. Considering PACSLAC-II behavioral cues for trained judges, significantly stronger reliance was found on the following cues when using the panoramic view: P1 (grimacing; Pearson–Filon *Z* = − 2.68, *p* < .05), P2 (tighter face; Pearson–Filon *Z* = 1.81, *p* < .05), P3 (pain expression; Pearson–Filon *Z* = 2.28, *p* < .05), and P5 (wincing; Pearson–Filon *Z* = − 2.68, *p* < .05). There were no significant difference in PACSLAC-II cue utilization for untrained judges when contrasting the profile view versus the panoramic view.

In summary, untrained coders tended to rate pain intensity higher during a physiotherapy examination when judging facial expressions from a profile view. They were also able to distinguish between baseline and pain segments. Untrained observers were also significantly more accurate when judging pain intensity from the profile than the front view. Based on comparisons of lens model analyses results, untrained observers tended to rely less on specific pain cues, compared to trained observers, particularly from the profile view.

## Discussion

In two studies, one based on fine-grained analysis of older adult participants’ nonverbal behavior by trained observers using two different observational techniques; the other based on a judgment study employing untrained observers, similar outcomes were observed. In “Study 1”, well-validated and rigorous behavioral observation techniques were both sensitive to the presence of pain, whether the visual image was from a direct frontal angle, and presumably optimal from the point of view of availability of the full array of information available in a behavioral expression, or viewed laterally (i.e., profile view). The mean differences between panoramic and lateral views were sufficiently small for both FACS-based and PACSLAC-II measures. In “Study 2”, which involved evaluation of untrained observers’ ratings relative to the standard set by FACS-based and PACSLAC-II measurement, the lateral view also differentiated pain-related from non-pain related segments as well as the panoramic view. Moreover, while untrained observers assigned higher pain intensity ratings to individuals undergoing a physiotherapy examination when viewing from a profile view, the profile view actually supported a superior correspondence between observers’ profile ratings and the direct behavioral index of pain expression relative to the panoramic view.

The particular finding of an advantage for the profile view over the panoramic view is counterintuitive. Although it is frequently assumed that the front view of the face is ideal for the coding of facial expressions of pain, and indeed for extracting the maximal degree of information from facial displays of any affective state, there are both theoretical and empirical reasons to question that assumption. Facial and other nonverbal expressions of affective states, such as pain, are evolved products resulting from the survival and reproductive advantages that derive from their ability to communicate internal states or probable future behavior (Fridlund 1994; de C. Williams 2002; de C. Williams and Kappesser 2018). As elements of a communicative process, both the ability to express pain intensity on the part of a sufferer and the ability to decode it on the part of observers have co-evolved in the environment of evolutionary adaptation (Prkachin et al. 2018; de C. Williams 2002). Arguably, pain-related behavior in that environment rarely occurred under conditions that resembled a videographer’s studio. To be truly efficient, an interpersonal communication system for pain (or other affective states) should enable effective interpersonal responses under a variety of less than ideal environmental conditions. With respect to pain, in particular, conceivable evolutionary scenarios such as injuries taking place by accident or in the course of agonistic encounters would rarely involve direct en-face observation by conspecifics. Thus, a natural process supporting effective nonverbal communication of pain in circumstances of varying visual information is plausible. It does not, however, provide a basis for expecting there to be an advantage to a visual angle that departs from face-on as the findings of the present study suggest.

Our findings strongly support the idea that there is no disadvantage and indeed may be an advantage to a lateral view. Reliability of frame-by-frame coding using the profile view ranges from satisfactory to comparable to having access to full view of the face. It will be important for future research to replicate these results with additional sets of coders. When it comes to observer judgments, where the coding is done in a clinical manner (i.e., without the aid of frame-by-frame video), there seem to be distinct advantages based on profile observations versus front of the face; more variance is accounted for in the differentiation of painful from non-painful situations. Moreover, the observers’ judgments had greater correspondence to systematic coding when these judgments were based on a profile view.

Lens model analyses comparing judge type (trained vs. untrained) suggest that trained coders rely more on specific behavioral cues (e.g., orbit tightening) than do untrained observers, from both the panoramic and profile views. In other words, it appears that, not surprisingly, the untrained observers relied more on the gestalt of the full expression than on specific codable facial actions. Lens model analyses comparing cue utilization based on view (panoramic vs. profile) suggest that both trained and untrained observers utilized the cue of eyes closing more from the profile view, and that untrained judges relied more on orbit tightening and levator movement when judging from a profile view. The advantage of profile-based observations may be occurring because there is less to see in a profile view, making it more likely that there will be a focus on areas around the eye and mouth where most pain expression tends to occur. It may also be the case that observers were attending to different cues depending on view. For instance, it may be easier to see changes at the corners/edges of the eyes and mouth, especially in faces with wrinkles. In support of this, our lens model analyses suggest that untrained observers relied significantly more on orbit tightening, levator action, and closing of the eyes (FACS-based coding) when using a profile view versus panoramic view. In recent literature, there is some evidence to suggest that accurate pain judgment relies mostly on frown lines (not typically visible from the side) and mouth movements (Roy et al. 2015). Future research, perhaps involving eye-tracking technology, could aid in clarifying some of the reasons behind the profile view advantage. Given that untrained observers rated pain intensity higher from a profile view *and* were more accurate when doing so, it may be that a profile view offers an opportunity to lessen underestimation bias in pain intensity judgments.

From a theoretical standpoint, the findings have implications for communications models of pain (Hadjistavropoulos and Craig 2002; Hadjistavropoulos et al. 2011; Prkachin and Craig 1995) in that they add specificity to the factors that influence observers’ ability to decode pain messages. These findings also have potential applications. Specifically, work on computer vision technology to monitor pain behaviors in clinical settings (e.g., among patients with severe dementia) is currently underway (Asgarian et al. 2017; Ashraf et al. 2017). For maximum efficiency and utility it is important for such systems to be capable of identifying pain behaviors from multiple angles of observation. Since algorithm development for such systems is based on the results of behavioral coding conducted by humans our findings have implications for machine learning development efforts. The high levels of validity and reliability demonstrated by human coders in this study suggest that machine systems being developed for the monitoring of pain behaviors in clinical settings have the potential of effectively identifying pain behaviors from both front and side views of the face.

From a clinical standpoint, nursing staff may sometimes observe pain behaviors from a profile view for practical reasons. It is useful to know that such profile observations would not compromise the ability to identify pain expressions. If our findings are replicated in future studies, it might even be useful to recommend profile pain expression observation in clinical settings at least under some circumstances.

In terms of limitations of this work, we note that (as reported in the "Procedure" section) while the vast majority of the movement protocol was well captured by our cameras, there was a brief moment where the participants’ face could not be captured (when they momentarily sat on the side of the bed). As such, the number of pain behaviors captured by our observational approaches may reflect small underestimations in the actual number of pain behaviors displayed. We also note that in this study, we did not assess chronicity of the pain conditions of our participants. The prevalence of chronic pain in older adults is high as a consequence of increased likelihood over time of developing disorders involving pain (e.g., osteoarthritis, musculoskeletal injury; Gibson 2003; Rice et al. 2016) and highest among long-term care residents (Won et al. 2004). Given that our community participants were a clinical pain population undergoing physiotherapy, it is reasonable to assume that most participants experienced acute exacerbations of chronic musculoskeletal pain. Future research could determine the generalizability of our results to acute pain populations.

## References

[CR1] AGS Panel on Persistent Pain in Older Persons (2002). The management of persistent pain in older persons. Journal of the American Geriatrics Society.

[CR2] Ammaturo DA, Hadjistavropoulos T, Williams J (2017). Pain in dementia: Use of observational pain assessment tools by people who are not health professionals. Pain Medicine.

[CR3] Asgarian, A., Ashraf, A. B., Fleet, D., & Taati, B. (2017). Subspace selection to suppress confounding source domain information in AAM transfer learning. In *The International Joint Conference on Biometrics (IJCB)*.

[CR4] Ashraf, A., Asgarian, A., Browne, E., Prkachin, K., Zhao, S., Budnarian, P. et al. (2017). Facial landmark detection for pain expression recognition in older adults. In *Poster presentation at the annual conference of the AGE WELL national centres of excellence (Winnipeg, MB)*.

[CR5] Ashraf, A., Browne, E., Taati, B., Hadjistavropoulos, T., Prkachin, K., Asgarian, A. et al. (2016). Computer vision-based facial expression analysis for recognizing pain in older adults. In *Poster presentation, annual conference of AGE-WELL network of centres of excellence (Montreal, Canada)*.

[CR6] Ashraf AB, Lucey S, Cohn JF, Chen T, Ambadar Z, Prkachin K, Solomon PE (2009). The painful face–pain expression recognition using active appearance models. Image and Vision Computing.

[CR7] Aubin M, Giguère A, Hadjistavropoulos T, Verreault R (2007). The systematic evaluation of instruments designed to assess pain in persons with limited ability to communicate. Pain Research and Management.

[CR8] Bartlett MS, Littlewort GC, Frank MG, Lee K (2014). Automatic decoding of facial movements reveals deceptive pain expressions. Current Biology.

[CR9] Brunswik E (1952). The conceptual framework of psychology. Psychological Bulletin.

[CR10] Chan S, Hadjistavropoulos T, Williams J, Lints-Martindale A (2014). Evidence-based development and initial validation of the pain assessment checklist for seniors with limited ability to communicate-II (PACSLAC-II). The Clinical Journal of Pain.

[CR11] Craig KD, Patrick CJ (1985). Facial expression during induced pain. Journal of Personality and Social Psychology.

[CR12] Craig KD, Prkachin KM, Grunau RVE, Turk DC, Melzack R (2001). The facial expression of pain. Handbook of pain assessment.

[CR13] de C. Williams, A. C. (2002). Facial expression of pain: An evolutionary account. *Behavioral and Brain Sciences,**25,* 439–445. 10.1017/s0140525x02000080 12879700

[CR14] de C. Williams, A. C., & Kappesser, J. (2018). Why do we care? Evolutionary mechanisms in the social dimension of pain. In T. Vervoort, K. Karos, Z. Trost, & K. M. Prkachin (Eds.), *Social and interpersonal dynamics in pain: We don’t suffer alone* (pp. 3–22). New York: Springer.

[CR15] Ekman P, Friesen WV (1978). Facial action coding system: Investigators’ guide.

[CR16] Fridlund AJ (1994). Human facial expression: an evolutionary view.

[CR17] Gagnon MM, Hadjistavropoulos T, MacNab YC (2017). Contextual influences on pain communication in couples with and without a partner with chronic pain. Pain.

[CR18] Gallant NL, Hadjistavropoulos T (2017). Experiencing pain in the presence of others: A structured experimental investigation of older adults. The Journal of Pain.

[CR100] Gibson SJ (2003). Pain and aging: The pain experience over the adult life span. Progress in Pain Research and Management.

[CR19] Hadjistavropoulos T, Browne ME, Prkachin KM, Taati B, Ashraf A, Mihailidis A (2018). Pain in severe dementia: A comparison of a fine-grained assessment approach to an observational checklist designed for clinical settings. European Journal of Pain.

[CR20] Hadjistavropoulos T, Craig KD (2002). A theoretical framework for understanding self- report and observational measures of pain: A communications model. Behavior Research and Therapy.

[CR21] Hadjistavropoulos T, Craig KD, Duck S, Cano A, Goubert L, Jackson PL (2011). A biopsychosocial formulation of pain communication. Psychological Bulletin.

[CR22] Hadjistavropoulos T, Herr K, Prkachin KM, Craig KD, Gibson SJ, Lukas A, Smith JH (2014). Pain assessment in elderly adults with dementia. The Lancet Neurology.

[CR23] Herr K (2011). Pain assessment strategies in older patients. The Journal of Pain.

[CR24] Herr K, Coyne P, McCaffery M, Manworren R, Merkel S (2011). Pain assessment in the patient unable to self-report: Position statement with clinical practice recommendations. Pain Management Nursing.

[CR25] Husebo BS, Strand LI, Moe-Nilssen R, Borge Husebo S, Aarsland D, Ljunggren AE (2008). Who suffers most? Dementia and pain in nursing home patients: A cross-sectional study. Journal of the American Medical Directors Association.

[CR26] Husebo BS, Strand LI, Moe-Nilssen R, Husebo SB, Ljunggren AE (2010). Pain in older persons with severe dementia. Psychometric properties of the Mobilization–Observation–Behavior–Intensity–Dementia (MOBID-2) pain scale in a clinical setting. Scandinavian Journal of Caring Sciences.

[CR27] Husebo BS, Strand LI, Moe-Nilssen R, Husebo SB, Snow AL, Ljunggren AE (2007). Mobilization-observation-behavior-intensity-dementia pain scale (MOBID): Development and validation of a nurse-administered pain assessment tool for use in dementia. Journal of Pain and Symptom Management.

[CR28] Kunz M, Karos K, Vervoort T, Vervoort T, Karos K, Trost Z, Prkachin K (2018). When, how, and why do we express pain?. Social and interpersonal dynamics in pain: We don’t suffer alone.

[CR29] Kunz M, Prkachin KM, Lautenbacher S (2013). Smiling in pain: Explorations of its social motives. Pain Research and Treatment.

[CR30] Kunz M, Scharmann S, Hemmeter U, Schepelmann K, Lautenbacher S (2007). The facial expression of pain in patients with dementia. PAIN®.

[CR31] Lints-Martindale AC, Hadjistavropoulos T, Barber B, Gibson SJ (2007). A psychophysical investigation of the facial action coding system as an index of pain variability among older adults with and without Alzheimer’s disease. Pain Medicine.

[CR32] Lints-Martindale AC, Hadjistavropoulos T, Lix LM, Thorpe L (2012). A comparative investigation of observational pain assessment tools for older adults with dementia. The Clinical Journal of Pain.

[CR33] Morris JN, Fries BE, Mehr DR, Hawes C, Phillips C, Mor V, Lipsitz LA (1994). MDS cognitive performance scale^©^. Journal of Gerontology.

[CR34] Noldus Information Technologies (2015). The observer XT 12.5.

[CR35] Pearson K, Filon LNG (1898). Mathematical contributions to the theory of evolution. IV. On the probable errors of frequency constants and on the influence of random selection on variation and correlation. Proceedings of the Royal Society of London.

[CR36] Prkachin KM (1992). The consistency of facial expressions of pain: A comparison across modalities. Pain.

[CR37] Prkachin KM, Browne ME, Kaseweter KA, Vervoort T, Karos K, Trost Z, Prkachin KM (2018). The spectrum of third-person pain: From observation to action. Social and interpersonal dynamics in pain: We don’t suffer alone.

[CR38] Prkachin KM, Craig KD (1995). Expressing pain: The communication and interpretation of facial pain signals. Journal of Nonverbal Behavior.

[CR39] Prkachin KM, Solomon PE (2008). The structure, reliability and validity of pain expression: Evidence from patients with shoulder pain. Pain.

[CR40] Rice ASC, Smith BH, Blyth FM (2016). Pain and the global burden of disease. Pain.

[CR41] Roy C, Blais C, Fiset D, Rainville P, Gosselin F (2015). Efficient information for recognizing pain in facial expressions. European Journal of Pain.

[CR42] Steiger JH (1980). Tests for comparing elements of a correlation matrix. Psychological Bulletin.

[CR43] Won A, Lapane K, Vallow S, Schein J, Morris J, Lipsitz L (2004). Persistent nonmalignant pain and analgesic prescribing patterns in elderly nursing home residents. Journal of the American Geriatrics Society.

